# Selective Dorsal Rhizotomy for Treatment of Hereditary Spastic Paraplegia-Associated Spasticity in 37 Patients

**DOI:** 10.7759/cureus.17690

**Published:** 2021-09-03

**Authors:** TS Park, Susan Joh, Deanna M Walter, Nicole L Meyer

**Affiliations:** 1 Pediatric Neurosurgery, Washington University School of Medicine, St. Louis Children's Hospital, St. Louis, USA

**Keywords:** hereditary spastic paraplegia, selective dorsal rhizotomy, spasticity, ambulation, genetics

## Abstract

Background

A limited number of publications have described a reduction of spasticity associated with hereditary spastic paraplegia (HSP) after selective dorsal rhizotomy (SDR). Typically, the SDR procedure is performed on patients with spastic cerebral palsy to remove spasticity and to help these patients with ambulatory function. Whether SDR has similar effects on HSP patients, requires further investigation. Thus, we are providing a personal experience of the effects of SDR on this specific cohort of patients.

Objectives

To examine the safety of SDR, changes in spasticity, and ambulatory function after SDR on patients with HSP.

Methods

The Institutional Review Board of Washington University School of Medicine approved this study (#201704003). A total of 37 children and adults received SDR for the treatment of HSP-associated spasticity between 1988 and 2021. SDR was performed through an L1 laminectomy, as we previously described in an earlier publication. The patients took part in the follow-up examination either in-person or by email. The follow-up focused on the patients’ motor functions (primarily ambulation), adverse effects of SDR, and orthopedic treatments after SDR.

Results

Of the total 37 patients who participated in this study, 46% were female and 54% were male. The age range of when HSP was diagnosed was one month to 34 years. Six of the patients’ diagnoses were made, based on the family history of HSP in six patients and the remaining 31 patients’ diagnoses were confirmed by genetic tests. The most common genetic mutations were SPG4 and SPG3A. Of the patients with positive genetic tests, 40% had no family history of HSP. SDR was performed at the age of 2 to 45 years (mean: 14.7 years). The follow-up period ranged from 0 to 33 years (mean: 3.8 years). One patient developed a spinal fluid leak requiring surgical repair. Two patients reported mild numbness in parts of the lower limbs. Spasticity was removed in 33 patients (89%). Four patients (11%) experienced a return in spasticity. Regarding ambulatory function, 11% of patients reported a decline in function. Two patients walked independently before surgery but declined, requiring a wheelchair eight years and seven years, respectively, after surgery for each patient. In contrast, 16% saw an improvement in ambulatory function, improving from walking with a walker to walking independently. The remaining 73% of patients maintained their level of ambulation. These two groups of patients showed improvement in other motor functions and independence.

Conclusions

The present analysis suggests the potential role of SDR in the management of spasticity in HSP patients. We found no sign of SDR being a direct cause of deleterious effects on patients with HSP.

## Introduction

Hereditary spastic paraplegia (HSP) is comprised of a group of genetically and clinically heterogeneous neurodegenerative disorders [1]. The pathological hallmark of the disorder is the presence of axon degeneration of corticospinal tracts in the distal spinal cord [2]. Currently, there are no treatments that can prevent or reverse these pathological changes. The main clinical signs of this disorder are motor weakness and spasticity, which are predominantly observed in the lower limbs.

The current treatment of HSP is symptomatic and includes physical therapy and spasticity management. For spasticity management, anti-spasticity oral medications, Botox injections, intrathecal baclofen infusion, and selective dorsal rhizotomy (SDR) have been employed [3]. Earlier reports described SDR reducing spasticity and improving motor functions in a small series of HSP patients [4,5]. The present report details clinical information of 37 children and adults who received SDR at our institution between 1988 and 2021.

## Materials and methods

The Institutional Review Board of Washington University School of Medicine approved this study (#201704003). We obtained consent either directly from patients or their parents or guardians. At the Center for Cerebral Palsy Spasticity, over 4,500 patients have been treated for spasticity by the means of SDR, and only 37 of those patients were diagnosed with HSP. The present study includes the total of all 37 children and adults (ages: 3-49) who received the SDR procedure for the treatment of HSP-associated spasticity between 1988 and 2021. SDR was performed through an L1 laminectomy as previously reported [6]. About 65% of dorsal rootlets at the L1-S1 level were divided among each patient. We gathered their contact information from our database and medical records. The patients were followed up with either an in-person examination or by email.

For either follow-up method, several questions were asked about the patient. Questions about body function included level of ambulatory function (independent, walks with a walker, walks with crutches, or relies on a wheelchair) and sensory loss on any part of the lower limbs. Further questions about the perception of health, perception of SDR (whether they feel it improved the patient's overall quality of living), and negative effects of SDR were asked. Information about post-SDR orthopedic surgeries was obtained as orthopedic surgery is part of the treatment process for many patients with SDR. For those who were emailed, an initial survey was sent, then other follow-up questions were sent as needed.

Demographic information was obtained from our medical records. Demographic information included the date of birth, age at the time of this study, age at surgery, follow-up period, and gender of the patient.

Each patient’s information on their diagnosis of the specific gene mutation known to cause HSP and family history of mutation or diagnosis were found through our medical records. Patients were contacted through email or phone to confirm the genetic tests and results of HSP. The specific gene mutation was confirmed by contacting patients without a record of the specific gene mutation.

## Results

Of the 37 patients, the gender distribution was 44% female and 56% male. The age at the time of diagnosis ranged from one month to 34 years, with a majority of patients diagnosed before the age of five years. Three patients were diagnosed in adulthood. Another three patients recently received SDR and the follow-up period at the time of this study was short. These three patients are still currently being monitored. Six patients (17%) were diagnosed based on family history of HSP. These patients did not have genetic tests. The remaining 31 patients (83%) were diagnosed with positive genetic test results. The most common gene mutations were SPG4 and SPG3A. Of the 30 patients who had positive genetic tests, 40% had no family member with HSP (Table 1). Brain MRI was obtained in twenty patients. MRI was normal in 80% of the patients who had MRI. Three patients showed mild periventricular leukomalacia and one patient showed white matter heterotopia.

**Table 1:** Summary of hereditary spastic paraplegia patients treated with selective dorsal rhizotomy. SDR - selective dorsal rhizotomy

Patient	Gender	Age of Diagnosis (Year)	Genetic Type	Family history	Age at SDR (years)	Follow-Up (years)	Pre-SDR Ambulation	Post-SDR Ambulation
1	F	34	SPG3A	Yes	11.4	32.92	Independent	Independent
2	F	2	SPG3A	Yes	2.6	8.50	Independent	Independent
3	M	2	SPG4	Yes	2.8	8.42	Used Walker	Wheelchair
4	F	2	SPG11	No	2.7	7.50	Independent	Uses walker
5	M	6	No genetic test	Yes	16.7	7.33	Independent	Independent
6	M	7	No genetic test	Yes	10.7	6.67	Used Walker	Independent
7	F	9	SPG4	No	3.2	6.08	Used Walker	Uses walker
8	F	9	SPG10	No	8.0	5.58	Independent	Independent
9	F	1	No genetic test	Yes	43.8	5.50	Independent	Independent
10	M	15	SPG6	No	15.2	5.42	Used Walker	Independent
11	F	2	No genetic test	Yes	16.5	5.25	Independent	Independent
12	M	6	SPG 4	No	11.3	3.92	Independent	Independent
13	M	4	SPG10	No	5.0	3.67	Independent	Independent
14	M	15	SPG3A	Yes	43.4	3.42	Independent	Independent
15	M	2	SPG3A	No	7.1	2.67	Used Walker	Uses walker
16	M	1	SPG3A	No	2.6	2.67	Independent	Independent
17	F	1	SPG54	Yes	3.6	2.50	Used Walker	Uses Walker
18	M	1 month	No genetic test	Yes	44.8	2.33	Used Walker	Independent
19	M	7	SPG30	No	9.6	2.17	Independent	Independent
20	M	5	SPG3A	Yes	36.8	2.17	Independent	Independent
21	M	20	No genetic test	Yes	39.3	2.08	Used Walker	Uses walker
22	M	2	SPG7	Yes	5.4	1.75	Used Walker	Wheelchair
23	M	2	SPG7	Yes	7.1	1.75	Independent	Independent
24	F	1	SPG4	Yes	17.4	1.75	Used Walker	Uses walker
25	M	1	No genetic test	Yes	29.7	1.58	Used Walker	Independent
26	F	2	SPG4	No	5.2	1.50	Used Walker	Independent
27	M	2	SPG4	Yes	5.2	1.50	Independent	Uses Cane
28	F	15	SPG15	No	18.4	1.42	Independent	Independent
29	F	2	SPG4	Yes	2.5	0.92	Used Walker	Uses Walker
30	F	1	SPG3A	No	2.7	0.67	Used Walker	Uses Walker
31	M	8 months	SPG4	Yes	3.5	0.58	Used Walker	Uses Walker
32	F	3	SPG4	No	7.3	0.58	Used Walker	Uses Walker
33	M	3	SPG4	Yes	5.5	0.58	Independent	Independent
34	F	31	SPG4	Yes	46.1	0.33	Independent indoors	Independent indoors
35	F	14	SPG11	Yes	22.5	0.08	Independent	Uses Walker
36	M	14	SPG11	Yes	20.4	0.08	Independent	Uses Walker
37	F	5	SPG56	No	8.6	0.00	Independent	Independent

Regarding the age at the time of SDR, 10 patients (27%) were adults, aged between 18 and 46 years. Twenty-seven patients (73%) were children, including 14 children between 2 and 5 years of age. The patient's progression was followed for four months to 31 years. Twenty-two patients were followed for a minimum of 12 months. Fourteen patients had a follow-up period ranging from 2 to 31 years. SDR removed spasticity in 33 patients (89%). Four patients (11%) had recurrent or residual spasticity which was less severe than the spasticity before surgery. Of these four patients with recurrent or residual spasticity, three children had SPG4 or SPG10, and one adult had SPG3A genetic mutations. The time period in which the spasticity returned was 4, 6, 7, and 18 months.

Regarding ambulatory function before SDR, 21 patients (57%) walked independently, and 16 patients (43%) walked with a walker (Table 1). One child was non-ambulant but could take steps in a walker with support. The goal of surgery for this child was to improve sitting and comfort.

After SDR, 26 patients (70%) maintained their level of ambulation before and after surgery. Five patients (14%) improved their ambulation from walking with a walker to walking independently. Six patients (16%) deteriorated in their ability to walk. All of the patients who walked independently before surgery deteriorated to using a walker or cane; none required a wheelchair (Table 1). One child walked independently before and after SDR; later the child underwent a heel cord lengthening surgery at our center, which was followed by excessive foot dorsiflexion and knee crouching. The child now uses a walker and is the only child who was over-lengthened. However, we recommend orthopedic surgery to lengthen the hamstrings and heel cords for these patients. Those who improved or maintained the preoperative level of ambulation improved independence and quality of walking, sitting, and transition movements after surgery. Also, after the spasticity was reduced, patients were able to perform strengthening exercises.

Concerning sensory function after SDR, two patients experienced numbness in parts of the lower limbs. We did not examine vibratory sense in our patients before or after SDR.

Regarding orthopedic surgery, five patients received heel cord lengthening surgery before SDR. Another patient had an intrathecal baclofen pump placed. After SDR, eight patients received orthopedic surgery, i.e., heel cord and hamstring or adductor lengthening surgery.

## Discussion

Results of the present analysis suggest the potential role of SDR in spasticity management of HSP patients. SDR could remove or reduce HSP spasticity in all patients without deleterious effects on the clinical course of HSP. The reduction of spasticity accompanied improved ambulatory function.

The idea of performing SDR to treat HSP-related spasticity originated from the management of spasticity in cerebral palsy (CP) [7]. In patients with CP, spasticity is only harmful and has no proven benefit to leaving untreated. It continues to reduce neurological functions and musculoskeletal systems in children and adults. Spasticity inhibits motor movements, exercise, muscle flexibility, and longitudinal muscle growth during development [8,9]. Also, it contributes to the development of muscle contracture and bony deformities [8-10]. As patients age, spasticity causes “early aging” associated with loss of mobility in adulthood [11]. Spasticity associated with HSP may also have similar negative effects. While treating more than 4,600 CP patients with SDR since 1987, we found that SDR can remove CP spasticity permanently. After the surgery, spasticity management is unnecessary, and motor functions and quality of life improve. Moreover, patients can engage in strengthening exercises, which further improves motor functions.

Our first HSP patient underwent SDR surgery 33 years ago in 1988 (Video 1).

**Video 1:** Pre- and post-operative comparison of SDR Permission was given by the patient to display patient identifying information in the video.

At that time, genetic testing for HSP was not available. The patient was born at full term. With no family history of HSP or CP, she was diagnosed with spastic diplegia at 18 months of age. The cause of her CP was unknown but we proceeded to perform SDR on her at the age of 11 years. Prior to SDR, she had spastic weakness in the lower extremities; she walked independently with knee hyperextension, and excessive lateral trunk and pelvis movement. Since SDR, she has been free of spasticity. Currently, she walks independently and uses a cane at work for added stability. She has muscle pain in her back and quadriceps, with mild hypesthesia in the quadriceps. She lives with her husband and children. Her young daughter developed toe walking, prompting a genetic test for the daughter and mother. The genetic test revealed an SPG3 mutation in both individuals.

Because of the encouraging post-SDR course observed in the first patient and availability of specific genetic tests, we began to recommend SDR for HSP patients a decade ago. Our selection criteria for surgery are 1) “Uncomplicated” HSP diagnosed by a positive genetic test or a family history of a first-degree relative who is similarly affected; we do not recommend the surgery if HSP accompanies neurologic abnormalities such as seizures, cognitive impairment, or peripheral neuropathy. 2) Neurological abnormalities limited to lower extremity spastic weakness and bladder dysfunction; those with upper extremity spastic weakness are not recommended to undergo SDR. 3) Patient’s age of 2-50 years at the time of our evaluation. A caveat is that the currently available genetic tests do not include all genes known to cause HSP, and a negative genetic test does not exclude the diagnosis of HSP [1]. We have found that a repeat of a genetic test sometimes confirms the diagnosis. In a child born at full term who shows no abnormalities in brain magnetic resonance imaging (MRI) but has severe lateral trunk and pelvis movements when walking, HSP should be a differential diagnosis. When presented with a positive genetic test or family history, MRI is not mandatory. The MRI findings of HSP are nonspecific and are reported to include atrophy of the corpus callosum and spinal cord with a T2-signal intensity in the posterior limb of the internal capsule [12].

The age of onset and severity of disability varies greatly among members of the same family, in different families with the same genetic type, and between different genetic types of HSP [1]. Such complex clinical manifestations of HSP were also present in our patients, which makes it impossible to predict the true effects of the SDR procedure on the natural course of our patient’s HSP. However, we interpret the outcome to be positive based on our findings in our patients. In all except four patients, SDR removed spasticity.

## Conclusions

Our experience with 37patients suggests the potential role of SDR in the management of spasticity in HSP patients. SDR could remove or reduce HSP spasticity without deleterious effects on HSP. The reduction of spasticity accompanied improved ambulatory function. Other methods of spasticity management include anti-spasticity medication, Botox injection, and intrathecal baclofen infusion. The outcomes of each treatment are unknown, thus we cannot compare other treatments to SDR. Several patients reported enjoying an increase in quality of life after the SDR procedure.
